# Studies on the Q175 Knock-in Model of Huntington’s Disease Using Functional Imaging in Awake Mice: Evidence of Olfactory Dysfunction

**DOI:** 10.3389/fneur.2014.00094

**Published:** 2014-06-30

**Authors:** Craig F. Ferris, Praveen Kulkarni, Steven Toddes, Jason Yee, William Kenkel, Mark Nedelman

**Affiliations:** ^1^Center for Translational NeuroImaging, Northeastern University, Boston, MA, USA; ^2^Animal Imaging Research, Holden, MA, USA; ^3^Ekam Imaging, Boston, MA, USA

**Keywords:** BOLD fMRI, neural pathways, reward processing, awake animal imaging, mouse model of Huntington’s disease, huntingtin-associated protein 1, feeding behavior

## Abstract

Blood oxygen level dependent (BOLD) imaging in awake mice was used to identify differences in brain activity between wild-type, HETzQ175, and HOMzQ175 genotypes in response to the odor of almond. The study was designed to see how alterations in the *huntingtin* gene in a mouse model of Huntington’s disease would affect the perception and processing of almond odor, an evolutionarily conserved stimulus with high emotional and motivational valence. Moreover, the mice in this study were “odor naïve,” i.e., never having smelled almond or any nuts. Using a segmented, annotated MRI atlas of the mouse and computational analysis, 17 out of 116 brain regions were identified as responding differently to almond odor across genotypes. These regions included the glomerulus of the olfactory bulb, forebrain cortex, anterior cingulate, subiculum, and dentate gyrus of the hippocampus, and several areas of the hypothalamus. In many cases, these regions showed a gene-dose effect with HETzQ175 mice showing a reduction in brain activity from wild-type that is further reduced in HOMzQ175 mice. Conspicuously absent were any differences in brain activity in the caudate/putamen, thalamus, CA3, and CA1 of the hippocampus and much of the cortex. The glomerulus of the olfactory bulb in HOMzQ175 mice showed a reduced change in BOLD signal intensity in response to almond odor as compared to the other phenotypes suggesting a deficit in olfactory sensitivity.

## Introduction

Huntington’s disease (HD) is characterized by motor dysfunction and cognitive decline, and is caused by an autosomal dominant expansion of CAG repeats in the *huntingtin* (*HTT*) gene. HD patients often present with non-motor symptoms that include cognitive dysfunction (1, 2), affective disorders (3), and sleep and circadian rhythm disruptions, which can all precede the onset of motor dysfunction. In a recent imaging study, Enzi and coworkers reported deficits in reward processing in pre-manifest (near but not symptomatic) HD patients (4). Here, we use awake animal imaging to study the emotional and cognitive neural circuits involved in reward processing in a pre-symptomatic transgenic mouse model of HD. Indeed, with non-invasive ultra-high field, functional magnetic resonance imaging (fMRI) in awake animals, it is possible to image changes in brain activity across distributed, integrated neural circuits with high temporal and spatial resolution (5). When combined with the use of 3D segmented, annotated, brain atlases, and computational analysis it is possible to reconstruct the neural circuits involved in emotional and cognitive processes.

To this end, we report here, for the first time, the development and application of tools for blood oxygen level dependent (BOLD) imaging in awake mice without any invasive surgical preparation. This new technology was applied to the study of the zQ175 knock-in mice containing a human mutant (mHtt) allele with the expanded CAG repeat (~179 repeats) within the native mouse huntingtin gene (6). This animal model is representative of HD in humans from genetic, neural, and behavioral aspects. Both homozygous (HOM) and heterozygous (HET)zQ175 mice exhibit first signs of motor symptoms from 3 to 4 months of age and behavioral deficits accompanied by marked brain atrophy and brain metabolite changes by 8 months (6, 7). Indeed, the knock-in mouse models of HD show disease progression in the HET genotype as protracted and more subtle than HOM providing a better opportunity to identify biomarkers of early neuropathology prior to the onset of motor dysfunction (6–9).

The present studies were conducted on year old wild-type, HET zQ175, and HOM zQ175 mice. We show here that there are significant differences in brain activity in response to the smell of a highly desirable food – almond. In this study, all animals were “odor naïve” to this evolutionarily conserved stimulus (10) raising the following question. How has this single gene mutation altered the perception of this highly important odorant signal? The technology and approach used in this study addresses this question by showing that imaging awake mice is technically feasible and that the BOLD signal changes are robust and provide the means of differentiating brain activity between genotypes to an olfactory stimulus that carries high emotional and motivation valence. When these BOLD signal changes are registered to an MRI based, 3D segmented annotated mouse brain atlas, it is possible to identify the integrated neural circuits affected by this single gene mutation.

## Materials and Methods

### Animal care

Wild-type mice (C57B/L6J) (*n* = 5 male, 6 female) and knock-in zQ175 HET (*n* = 4 male, 6 female) and HOM (*n* = 5 male, 5 female) mice (courtesy of Psychogenics Inc., Tarrytown, NY, USA) were maintained on a 12:12 h light:dark cycle with a lights on at 07:00 hours. All mice from each phenotype were born between 16 and 18 August 2011 and scanned ~1 year later. Animals were allowed access to food and water *ad libitum*. All the mice were housed in groups of up to four per cage with mice of the same genotype and gender. Mice were cared for in accordance with the guidelines published in the Guide for the Care and Use of Laboratory Animals (National Institutes of Health Publications No. 85–23, Revised 1985) and adhered to the National Institutes of Health and the American Association for Laboratory Animal Science guidelines. The protocols used in this study were in compliance with the regulations of the Institutional Animal Care and Use Committee at Northeastern University.

### Awake mouse imaging system

**Figure 1:**
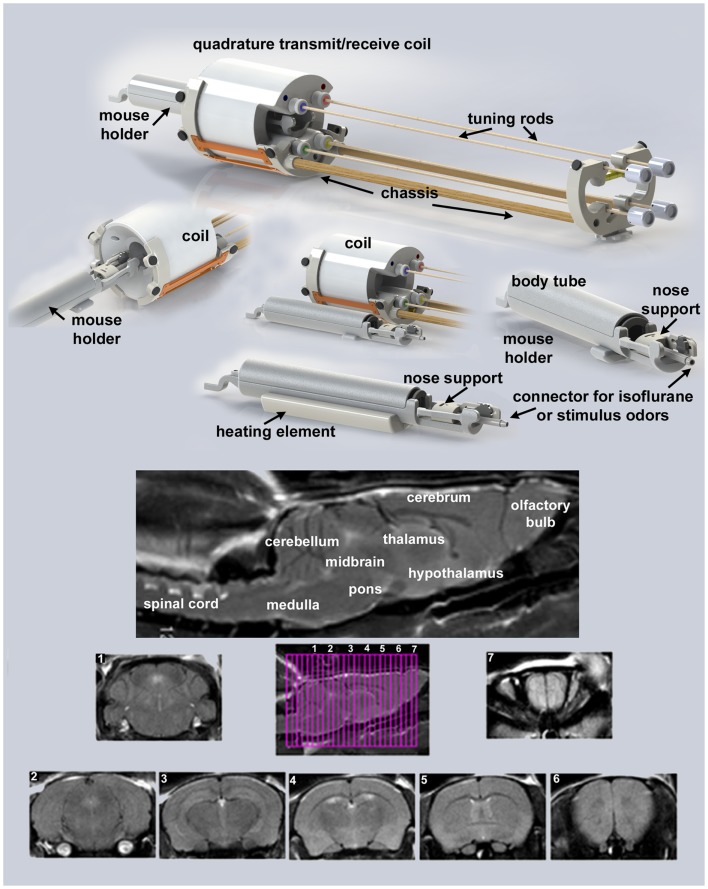
**Mouse imaging system**. Shown are the different components of the mouse imaging system. Below are sagittal and axial views of an awake mouse brain. Note the linearity along the *Z*-axis. The axial images taken from a 22-slice RARE sequence (0.6 mm thickness) demonstrate complete brain coverage from the olfactory bulbs to the brainstem. The mouse system was provided by *Animal Imaging Research*, Holden, MA, USA.

Presented in Figure 1 are the different components of the mouse imaging system showing a radiofrequency coil and MR compatible restraining system for imaging awake or anesthetized mice ranging in the size from 10 to 50 g (Animal Imaging Research, Holden, MA, USA). The quadrature transmit/receive volume coil (ID 38 mm) provides excellent anatomical resolution and signal–noise-ratio for voxel-based fMRI. The unique design of the holder essentially stabilizes the head in a cushion, minimizing any discomfort normally caused by ear bars and pressure points used to immobilize the head for awake animal imaging. The mouse holder can be inserted and withdrawn while the volume coil is positioned in the magnet greatly reducing the set-up time between studies. A movie showing the set-up of a mouse for awake imaging is available at http://www.youtube.com/watch?v=W5Jup13isqw.

**Figure 2:**
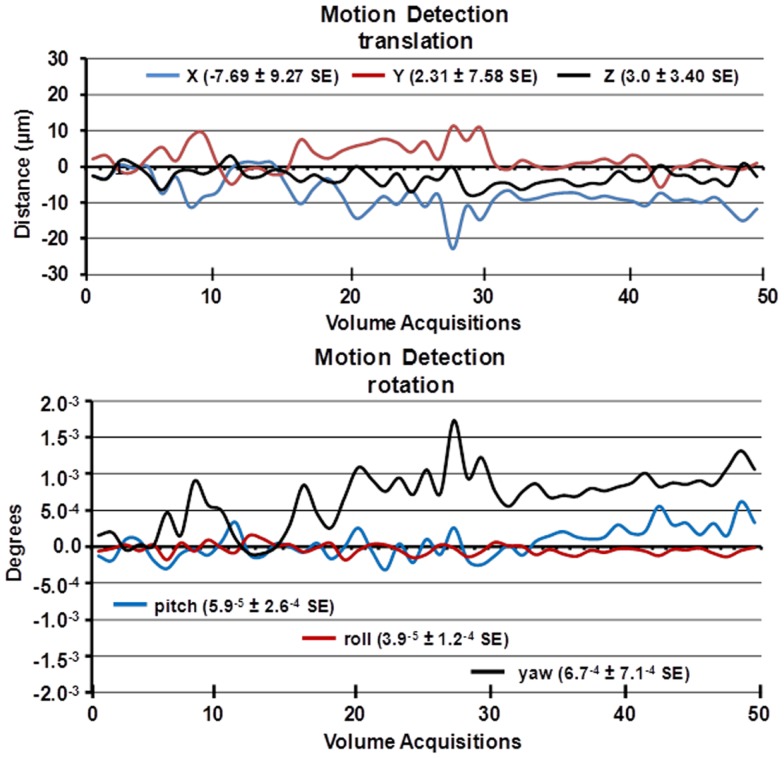
**Motion detection following acclimation**. Time-course date for motion detection from 29 awake mice imaged for 5 min is shown. Two and a half minute into the scanning session, mice were challenged with 5% carbon dioxide (see Figure 3). The data stability were estimated by a 3D rigid body model with 6 degrees of freedom for translational and rotational movement.

It should be noted, Desai and colleagues used BOLD imaging and optogenetics to study integrated neural circuits in awake mice (11). Their experimental design required that each animal be implanted with a head post. In our system, this is not necessary, eliminating the confound of surgery. Moreover, following acclimation it is possible to image multiple animals during an imaging session. The data presented on motion detection in Figure 2 were gathered from 29 mice imaged over a 8 h period. This was possible because the total scan time (tripilot, anatomy, and functional) was <15 min each. In addition, once the quadrature transmit/receive volume coil was position in the magnet, tuned, and matched, it was never moved or adjusted. One mouse holder (see Figure 1) was simply replaced with a second as noted above.

**Figure 3:**
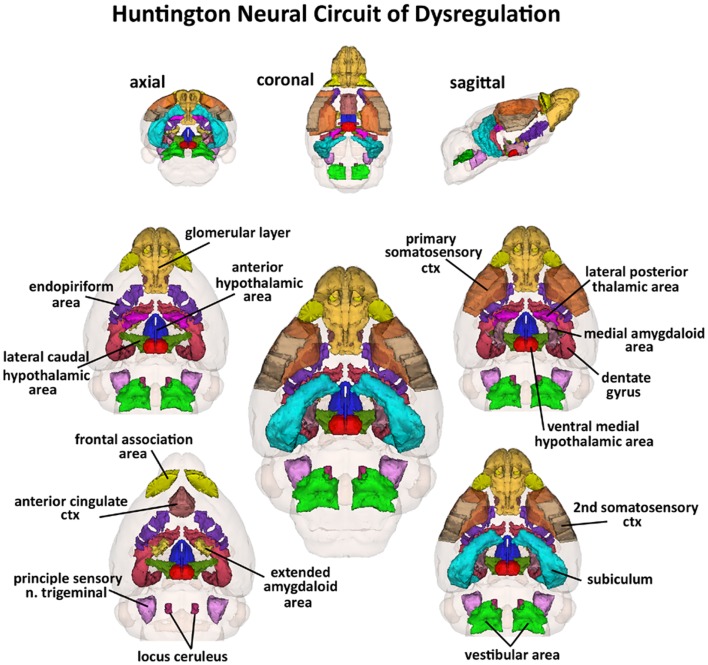
**Huntington neural circuit of dysregulation in response to almond odor**. The 17, color-coded and labeled 3D volumes shown above were identified from 116 brain areas in the mouse atlas as having a significantly different BOLD signal between genotypes in response to the odor of almond. These areas are listed in Table 1. The central image is a coronal view of a translucent shell of the brain showing the total composite and location of the different 3D volumes of interest. Surrounding this are different layers of the neural circuit showing a caudal (deepest) to dorsal perspective of the different brain volumes. The panels on the top show the neural circuit in different orthogonal directions.

One limitation in the mouse imaging system is the presentation of visual and auditory stimuli. While it is possible to inject drugs (IP, SC, and IV), electrically stimulate fore and hind paws, and run flexible fiber optics to the head through portals in the body tube, the eyes are not easily accessible to visual stimuli. The auditory canal of the ears is blocked by the cushioned head pad, dampening, if not eliminating, auditory stimuli. However, the presentation of odors is actually facilitated by the design of the head holder. The front incisors of the mouse are locked onto a bite bar by pulling the snout into a beveled nose cone. The cone is perforated so as not to restrict the flow of air from the nostrils or mouth. A hollow tube extends from the tip of the nose cone providing a route for administering volatile anesthetics, e.g., isoflurane, carbon dioxide gas as shown in Figure 3 or odors that carry emotional and/or cognitive messages. Furthermore, olfaction is an especially important sensory modality in rodents.

### Acclimation and motion detection

A week prior to the first imaging session, all animals were acclimated to the imaging system before scanning. Animals were secured into their holding system while anesthetized with 2–3% isoflurane. Following cessation of isoflurane, fully conscious mice were put into a “mock scanner” (a black box with a tape recording of MRI pulses) for 30 min for four consecutive days. Acclimation in awake animal imaging significantly reduces physiological effects of the autonomic nervous system including heart rate, respiration, corticosteroid levels, and motor movements (12) helping to improve contrast-to-noise and image quality. In these studies, motion artifact was greatly reduced as shown in the time-course data in Figure 2 as estimated by a 3D rigid body model with 6 degrees of freedom for translational and rotational movement. Data were collected from 29 awake mice imaged for 5 min. The average motion is <20 μ.

### Imaging acquisition and pulse sequence

Experiments were conducted using a Bruker Biospec 7.0 T/20-cm USR horizontal magnet (Bruker, Billerica, MA, USA) and a 20-G/cm magnetic field gradient insert (ID = 12 cm) capable of a 120-μs rise time (Bruker). At the beginning of each imaging session, a high-resolution anatomical data set was collected using the RARE pulse sequence (20 slice; 0.75 mm; FOV 2.5 cm; data matrix 256 × 256; TR 2.1 s; TE 12.4 ms; Effect TE 48 ms, NEX 6; 6.5 min acquisition time). Functional images were acquired using a multi-slice half Fourier acquisition single shot turbo spin echo (HASTE) pulse sequence. With this sequence it is possible to collect 20, 0.75 mm thick, axial slices in <6 s. With a FOV of 2.5 cm and a data matrix of 96 × 96, the in-plane pixel functional resolution for these studies was 260 μm^2^.

The major advantage to a spin echo pulse sequence is its tolerance to magnetic susceptibility and motion artifact. The 180° RF refocusing pulse corrects for the lack of field homogeneity, chemical shift, tissue artifacts, and magnetic susceptibility from static dephasing in BOLD imaging. The disadvantage is loss of signal contrast. The problem of sensitivity can be addressed with higher field strengths where the BOLD signal becomes a function of dynamic dephasing from diffusion of water at the level of the capillaries (13, 14). Using multi-slice, fast spin echo sequences the signal contrast with BOLD imaging is a function of T2 and not T2* at high field strengths. The extravascular signal surrounding capillary beds and small vessels is more reflective of the metabolic changes in brain parenchyma than signal from large draining veins helping to improve the localization of the signal changes (15). The BOLD signal is linear and reproducible at stimulus intervals of 1 s (16).

### Provocation paradigm – odor stimulant

Awake wild-type and transgenic mice were imaged for changes in BOLD signal intensity in response to the odor of almond (benzaldehyde), a stimulus to elicit the *innate reward response* (10). All animals were “odor naïve” to this evolutionarily conserved stimulus. We chose the almond odor because nuts are high in calories and convey greater valance as compared to the other odors. Moreover, the standard food chow is devoid of nuts, so laboratory bred mice and the mice used in these studies have no previous exposure to this food. In a recent study (10), brain activation maps from the odors of banana, rose, citrus, and almond were dramatically different. Almond but not the other odors activated the hippocampus, amygdala, and limbic cortex. In a serial dilution study for almond scent, we identified a threshold dilution of 100% benzaldehyde (1/10,000 v/v) that gives a significant and consistent pattern of brain activity. This threshold dilution of almond odor was used in this study. The time series for changes in BOLD signal following presentation of almond odor were analyzed using a repeated measures ANOVA followed by Fisher’s protected least significant difference to limit experiment-wise error when performing pairwise comparisons between genotypes.

### Data analysis

Images were aligned and registered to a 3D mouse brain atlas, which is segmented and labeled with 116 discrete anatomical regions (Ekam Solutions, Boston, MA, USA). The alignment process was facilitated by an interactive graphic user interface. The registration process involved translation, rotation, and scaling independently and in all three dimensions. Matrices that transformed each subject’s anatomy were used to embed each slice within the atlas. All pixel locations of anatomy that were transformed were tagged with major and minor regions in the atlas. This combination created a fully segmented representation of each subject within the atlas. The inverse transformation matrix [Ti]^−1^ for each subject (i) was also calculated.

Using voxel-based analysis, the percent change in BOLD signal for each independent voxel was averaged for all subjects. Each scanning session consisted of 70 data acquisitions (whole brain scans) with a period of 6 s each for a total lapse time of 420 s or 7 min. The control window was the first 25 scan repetitions (2.5 min baseline) while the odor stimulation window was 26–50 (min 2.5–5). Statistical *t*-tests were performed on each voxel (ca. 15,000 in number) of each subject within their original coordinate system with a baseline threshold of 2% BOLD change to account for normal fluctuation of BOLD signal in the awake rodent brain (17). As a result of the multiple *t*-test analyses performed, a false-positive detection controlling mechanism was introduced (18). This subsequent filter guaranteed that, on average, the false-positive detection rate was below our cutoff of 0.05. The *t*-test statistics used a 95% confidence level, two-tailed distributions, and heteroscedastic variance assumptions.

A composite image of the whole brain representing the average of all subjects was constructed for each group for ROI analyses, allowing us to look at each ROI separately to determine the BOLD change and the number of activated voxels in each ROI. Statistical comparisons of different image acquisitions are compared to baseline (see Table 1) using a non-parametric Kruskal–Wallis test statistic followed by a Mann-Whitney *U*-test.

**Table 1:** **Volume of activation in response to almond odor**.

Region of interest (ROI)	Wild-type			Heter zQ175			Homo zQ175			
	Med	Max	Min	Med	Max	Min	Med	Max	Min	*p* Value
Medial amygdaloid area	4	20	0	2	10	0	0	0	0	0.002
Ventral medial hypothalamic area	7	68	0	13	33	0	0	0	0	0.004
Frontal association ctx	10	23	3	0	14	0	0	11	0	0.006
Subiculum	3	27	0	1	4	0	0	2	0	0.008
Anterior hypothalamic area	3	27	0	4	14	0	0	0	0	0.008
Vestibular area	8	62	0	0	8	0	0	3	0	0.009
Endopiriform area	4	21	0	0	3	0	0	0	0	0.011
Extended amydala	1	11	0	0	3	0	0	0	0	0.012
Dentate gyrus	6	32	0	1	7	0	0	4	0	0.016
Anterior cingulate area	4	28	0	0	8	0	0	3	0	0.026
Lateral caudal hypothalamic area	4	39	0	4	25	0	0	11	0	0.026
Lateral posterior thalamic area	3	27	0	0	0	0	0	33	0	0.027
Primary somatosensory ctx	4	21	2	4	18	0	0.5	5	0	0.03
Secondary somatosensory ctx	1	14	0	3	6	1	0	4	0	0.032
Glomerular layer	6	29	1	3	40	1	0.5	10	0	0.044
Locus ceruleus	20	50	0	0	31	0	0	33	0	0.045
Principal sensory nucleus trigeminal	1	21	0	1	6	0	0	1	0	0.048
Medial mammillary area	23	82	0	3	31	0	0	35	0	0.059

*Shown is a truncated list of 116 brain areas and their median (med), maximum (max), and minimum (min) number of voxels as a percentage of the total ROI volume (i.e., number of voxels activated, divided by the total number of voxels in the 3D volume of interest, times 100) for wild-type, HET, and HOMzQ175 mice following exposure to odor of almond. These volumes of activation for each brain region across genotypes were analyzed using a Newman–Keuls multiple comparisons test statistic and rank order for their significance. *p* Values are presented on the far right column. The shaded gray columns highlight the median number of voxels for each ROI and each genotype to aid in visual comparisons*.

### Normalization of volume of activation

The differences BOLD signal change in wild-type, HETzQ175, and HOMzQ175 mice are reported in terms of volume of activation or number of voxels per region of interest (ROI) or brain area. In this study, the brain size across phenotypes can be significantly different (see Table 2) from each other hence the volume of activation is normalized to volume of ROI. By normalizing to volume of activation, we can compare across different phenotypes or within group among different regions. Normalized volume of activation was computed using following formula.

$$\begin{array}{llll} & \text{Normalized}\;\text{number}\;\text{of}\;\text{voxels}\;\text{in}\;\text{ROI}\; & & \;\\ & \;=\frac{\text{Number}\;\text{of}\;\text{activated}\;\text{voxels}\;\text{in}\;\text{ROI}\times 100}{\text{Total}\;\text{number}\;\text{of}\;\text{voxels}\;\text{in}\;\text{ROI}}.\; & & \; \end{array}$$

**Table 2:** **Brain volumes**.

Region of interest (ROI)	Wild-type			Heter zQ175			Homo zQ175			
	$\bar{X}$ Vol mm^3^	$\bar{X}$ Vox #	SE	$\bar{X}$ Vol mm^3^	$\bar{X}$ Vox #	SE	$\bar{X}$ Vol mm^3^	$\bar{X}$ Vox #	SE	*p* Value
Caudate putamen	17.6	1171	102.9	19.6	1304	93.1	14.8	387	93.8	0.03
Caudal piriform ctx	5.9	392	35.7	6.5	435	31.5	4.9	325	33.5	0.03
Endopiriform area	1.0	67	7.6	1.2	78	6.5	0.8	54	6.4	0.03
Anterior cingulate area	2.8	186	18.5	3.2	213	16.3	2.4	158	16.6	0.03
Fimbria hippocampus	1.8	123	11.8	2.1	139	10.3	1.5	102	11.4	0.03
Globus pallidus	2.7	180	18.0	3.0	2CI1	15.1	2.2	147	15.4	0.03
Primary somatosensory ctx	20.7	1381	127.2	22.9	1528	109.8	17.4	1158	116.0	0.03
Superior colliculus	8.3	551	48.4	9.3	618	43.3	6.9	458	43.7	0.03
Median raphe area	1.3	86	7.2	1.5	97	6.8	1.1	73	6.2	0.03
Substantia nigra	1.6	109	10.1	1.8	119	7.8	1.4	90	9.0	0.03
Parietal ctx	0.5	31	2.6	0.6	37	3.4	0.3	23	3.1	0.03
Prelimbic ctx	1.7	112	10.9	1.9	126	9.3	1.4	96	9.8	0.03
CA1 hippocampus	8.0	536	47.6	8.9	590	42.2	6.8	454	43.4	0.03
Entorhinal ctx	16.0	1066	93.1	17.7	1180	82.2	13.5	303	93.8	0.04
Olfactory tubercles	1.8	120	11.5	2.0	136	11.5	1.5	99	10.4	0.04
Ventral medial hypothalamic area	0.7	43	4.6	0.9	57	4.4	0.6	39	4.3	0.04
Medial geniculate	1.3	87	8.3	1.5	97	6.6	1.1	72	6.7	0.04
Retrosplenial caudal ctx	4.3	285	25.4	4.7	312	23.4	3.5	233	24.2	0.04
Ventral pallidum	1.9	128	13.2	2.2	144	11.5	1.6	107	11.7	0.04
Auditory ctx	3.8	256	24.2	4.4	290	20.4	3.2	216	23.0	0.04
Medial amygdaloid area	2.0	132	12.7	2.2	149	11.2	1.7	111	10.7	0.04
Dentate gyrus	6.3	420	38.4	6.9	460	34.0	5.2	347	37.4	0.04
Lateral geniculate	0.7	46	4.2	0.8	52	4.1	0.5	36	3.7	0.04
Basal amygdaloid area	3.1	206	20.2	3.5	230	18.2	2.6	173	17.8	0.04
Secondary somatosensory ctx	5.6	370	34.1	6.1	407	30.9	4.6	308	31.5	0.04
Lateral rostral hypothalamic area	2.9	191	17.3	3.2	214	15.3	2.4	160	17.1	0.04
Periaqueductal gray	5.3	352	32.2	5.8	388	28.9	4.4	296	28.5	0.04
Central amygdaloid area	1.8	122	11.4	2.0	133	9.7	1.5	103	10.3	0.04
Anterior hypothalamic area	2.3	152	13.1	2.5	169	11.3	2.0	131	12.1	0.04
Insular rostral ctx	5.9	392	34.4	6.5	431	31.6	4.9	326	32.7	0.04
Mesencephalic reticular formation	6.9	458	42.0	7.5	503	34.3	5.7	383	37.3	0.04
Ventral tegmental area	0.6	39	4.4	0.6	42	2.9	0.5	31	3.5	0.04
Subiculum	7.2	478	44.3	7.9	525	37.4	6.0	399	33.1	0.04
Ventral thalamic area	4.9	328	28.6	5.5	364	26.9	4.2	278	27.5	0.04
Inferior colliculus	6.5	436	40.8	7.2	482	34.2	5.6	370	36.6	0.05
Orbital ctx	5.5	367	33.2	6.1	407	29.6	4.7	311	30.4	0.05
CAS hippocampus	3.4	225	21.9	3.8	251	19.1	2.8	185	20.1	0.05
Rostral piriform ctx	8.1	540	47.9	8.9	590	42.8	6.7	443	47.0	0.05
Visual 1 ctx	13.6	303	82.6	14.7	378	69.4	11.6	773	75.9	0.05
Dorsal raphe	0.6	37	3.5	0.6	40	3.0	0.5	31	3.0	0.05
Primary motor ctx	5.1	337	32.6	5.7	377	30.6	4.3	287	31.7	0.05
Paraventricular hypothalamic area	0.2	10	1.4	0.2	12	1.1	0.1	8	1.2	0.05
Pituitary	0.9	57	6.4	1.0	68	5.4	0.7	48	5.4	0.06

*Shown is a truncated list of 116 brain areas and their mean volume (cubic millimeter), mean voxel number (#) with standard error (SE) for wild-type, HET, and HOMzQ175 mice. These brain volumes for each ROI for each genotype were analyzed using a Newman–Keuls multiple comparisons test statistic and rank order for their significance. *p* Values are presented on the far right column. The shaded gray columns highlight the mean volume for each ROI and each genotype to aid in visual comparisons*.

### Calculating the volumes of different brain areas

The volume of each brain area (ROI) was determined from the high-resolution anatomical scan taken at the beginning of each scanning session for each subject. The 3D segmented atlas provides the precise number of voxels (3D pixels) that combine to fill the volume of each of the 116 ROIs or brain regions. The dimensions of each voxel are calculated from the slice thickness (0.75 mm), Voxel width (FOV in X direction/Number of voxels in X direction) and Voxel height (FOV in Y direction/Number of voxels in Y direction) using the formula – Volume of voxel = voxel width × voxel height × slice thickness [ca. 0.097 mm × 0.097 mm × 0.750 mm = 0.00706 mm^3^]. Total number of voxels in each ROI were multiplied by volume of voxel to compute total volume of brain region.

### Carbon dioxide challenge

To assess the strength of the BOLD signal in mice using HASTE and to further characterize any differences between wild-type (*n* = 7), HETzQ175 (*n* = 7), and HOMzQ175 (*n* = 10) mice in terms of cerebrovascular reactivity animals were challenged with a 5% CO_2_ as a stimulus for a surrogate BOLD response. Carbon dioxide causes a direct relaxation of cerebrovascular smooth muscle, causing a passive dilation with a subsequent increase in cerebral blood flow. To this end, mice were imaged for a total of 5 min with presentation of 5% CO_2_ in ambient air at 2.5 min into the scanning session. Data were analyzed using a repeated measures ANOVA followed by Fisher’s protected least significant difference to limit experiment-wise error when performing pairwise comparisons between genotypes.

## Results

Shown in Table 1 are the positive BOLD signal changes represented as a percentage of the total ROI volume (i.e., number of voxels activated, divided by the total number of voxels in the 3D volume of interest, times 100) for wild-type, HET, and HOMzQ175 mice following exposure to odor of almond. The brain areas are rank order for their significance and are truncated from a larger list of 116 regions of activation (for complete list see Table S1 in Supplementary Material). The multiple comparison analysis showed 17 areas to be significantly different across conditions. Using the mouse brain atlas, these 17 brain areas can be reconstructed into a 3D map as shown in Figure 3. This 3D presentation is the putative neural circuit affected by the huntingtin gene mutation in response to the odor of almond.

**Figure 4:**
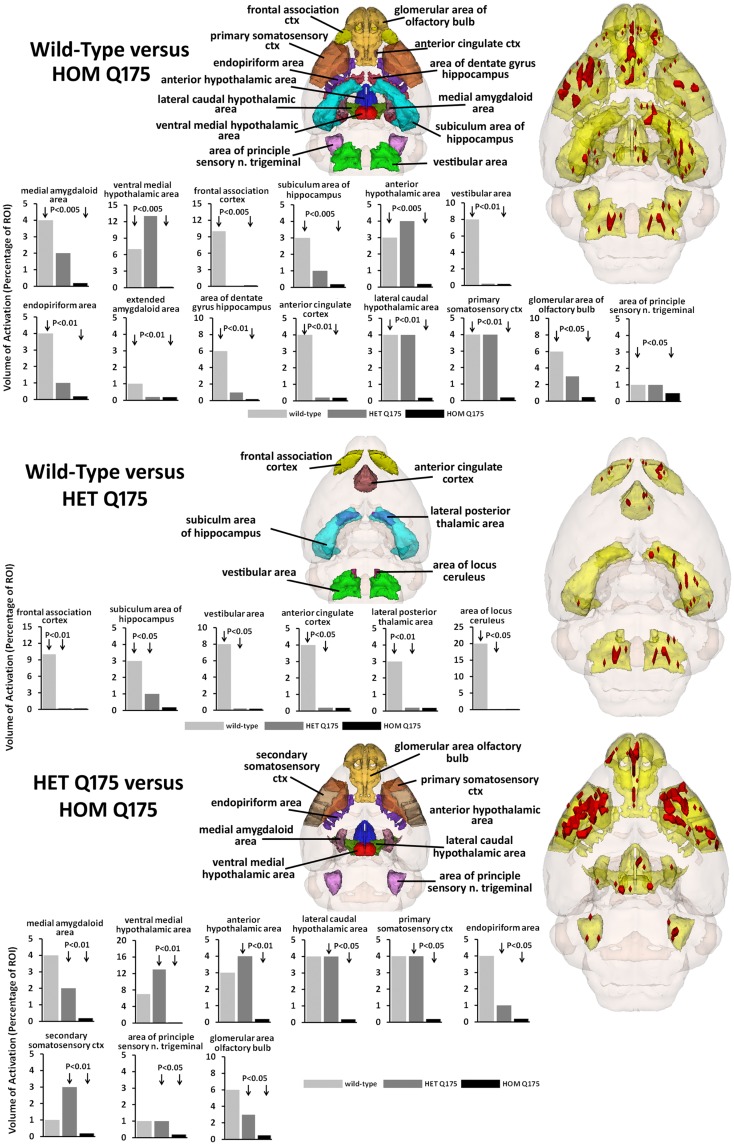
**Gene-dose effect in response to almond odor**. The figure is parsed into three panels based on the significant differences between wild-type and HOMzQ175 (top panel), wild-type and HETzQ175 (middle panel), and HETzQ175 and HOMzQ175 (lower panel). Areas that are significantly different for each comparison are presented as 3D color-coded and labeled maps above with the individual bars graphs for each brain area below. These 3D color-coded volumes are coalesced into a single yellow volume to the right of each panel showing the location of the average, significant change in BOLD signal (red). These 3D activation maps are data taken from wild-type (*n* = 9) for the top and middle panels. The 3D activation map in the bottom panel is data taken from HETzQ175 (*n* = 9). The bar graphs show the volume of activation as a percentage of the brain area volume for each statistical comparison using Mann-Whitney *U*-test.

The odor of almond elicits an activation pattern across wild-type, HET, and HOMzQ175 mice that suggests a gene-dose effect. The statistical comparisons between conditions and their composite pattern of activation are shown in Figure 4. The bar graphs show the volume of activation as a percentage of the ROI volume for each comparison. The areas of significance are shown in the segmented, 3D color-coded and labeled image above. These 3D areas are coalesced into a single yellow volume to the right showing the location of the average, significant change in BOLD signal. The *post hoc* analysis shows wild-type to have 14 brain areas that differed in activation from HOMzQ175. These include the forebrain cortical areas (primary somatosensory, frontal association, and anterior cingulate cortices), the hypothalamus (anterior, ventral medial, and lateral caudal areas), the dentate, and subiculum of the hippocampus and the glomerular layer of the olfactory bulbs. When wild-type is compared to HETzQ175 only six areas show a difference in BOLD activation. The frontal association and anterior cingulate cortices, subiculum, and vestibular areas are included showing that both HOM and HETzQ175 differ from wild-type in the activation of these brain areas in response to the odor of almond. HETzQ175 mice show nine brain area that differs from HOMzQ175. Included are all hypothalamic areas, both primary and secondary somatosensory cortices and glomerular layer of the olfactory bulb.

**Figure 5:**
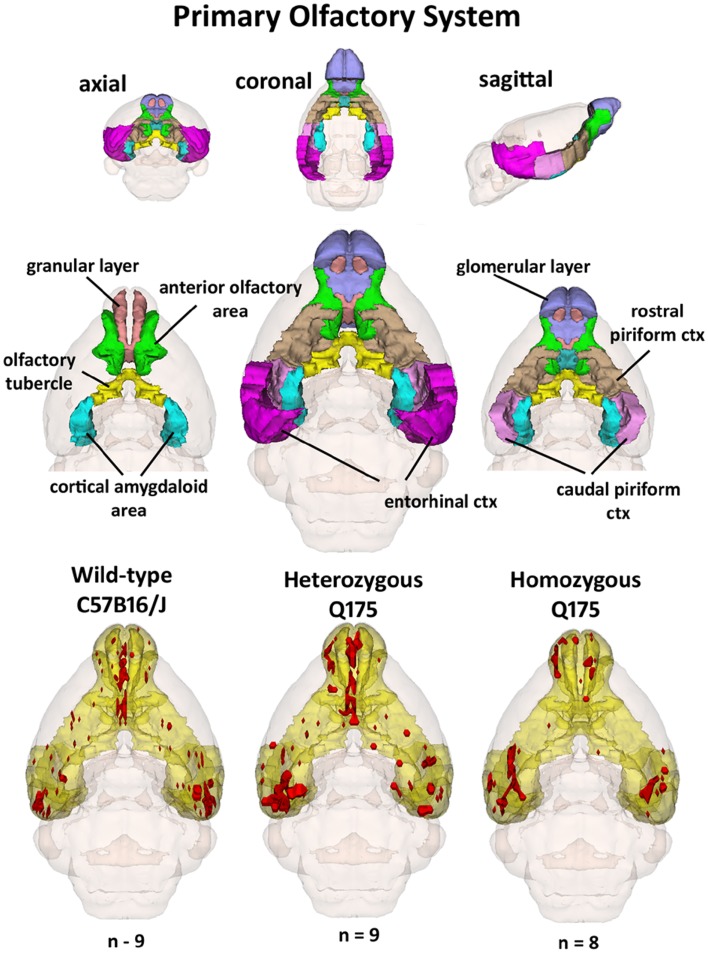
**3D activation maps of the primary olfactory system**. Shown is a 3D color representation of the different brain areas comprising the primary olfactory system. The layout is similar to that described in Figure 4. In the three illustrations below, these brain areas are coalesced into a single yellow volume. The red shows the location of the average, significant increase in BOLD signal in response to the presentation of almond odor for each genotype.

**Figure 6:**
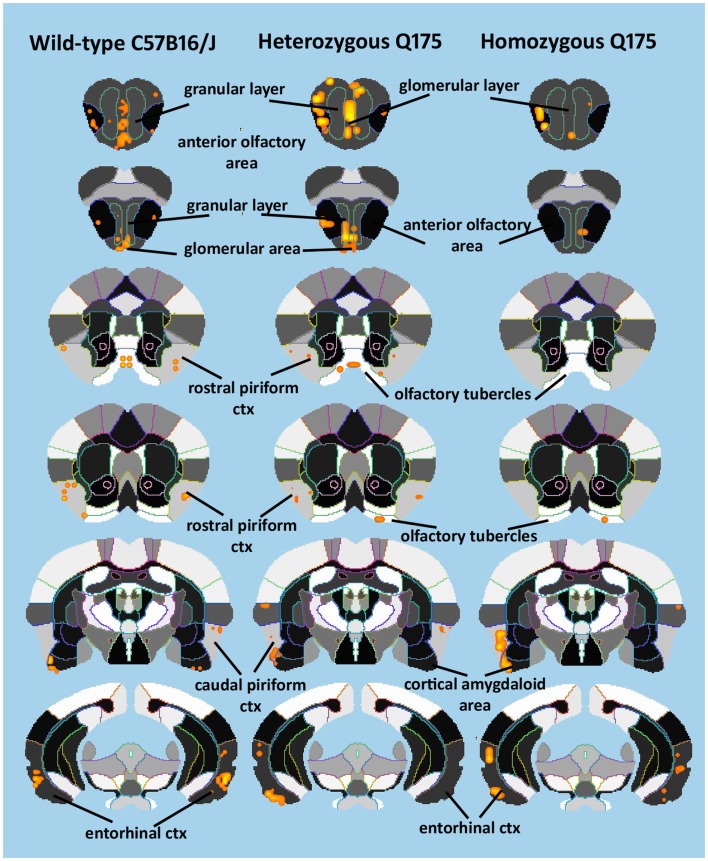
**2D activation maps of the primary olfactory system**. Shown are rostral (top) to caudal (bottom) axial sections taken from the mouse brain atlas and the location of the average, significant increase in BOLD signal for each genotype in response to the odor of almond. These are the same data shown in figure but with a 2D perspective to shown the signal location with the 3D volume.

Since the provocation paradigm for these patterns of brain activation was the odor of almond, the primary olfactory system was reconstructed in 3D as shown in Figure 5. Shown below are the 3D activation maps for each of the genotypes. As in Figure 4 the red depicts the localization of the average significant change in BOLD signal change for wild-type, HETzQ175, and HOMzQ175 mice. These same data are presented as activation maps in 2D axial sections shown in Figure 6. The most conspicuous difference between genotypes is the absence of BOLD activation in the glomerular layer of the olfactory bulb confirmed in Table 1.

**Figure 7:**
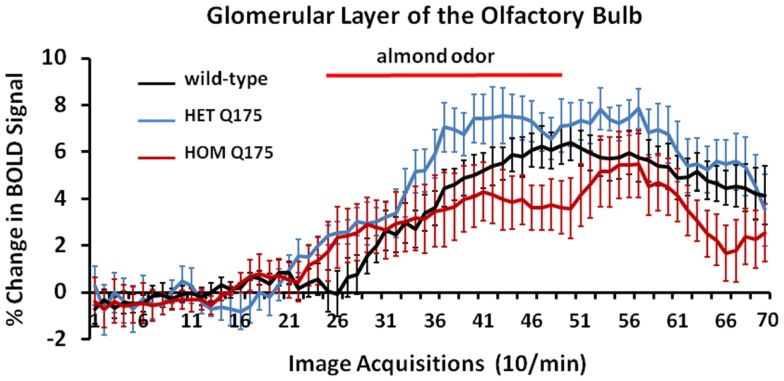
**Blood oxygen level dependent signal change over time in response to almond odor**. Shown are the changes in the BOLD signal in the glomerulus of the olfactory bulb for each genotype in response to the presentation of almond odor following a 2 min baseline of 20 image acquisitions. While the time series are similar in that each genotype shows a comparable baseline and onset of activation there is a significant difference between the time series and an interaction between the time series and genotypes as shown in the two-way repeated measures ANOVA. Each time point is the mean BOLD signal ± SEM.

Shown in Figure 7 are time-course data depicting the change in BOLD signal intensity in the glomerular layer of the olfactory bulb for wild-type, HETzQ175, and HOMzQ175 mice. Each mouse in the wild-type (*n* = 9) and HETzQ175 (*n* = 9) showed a significant change in BOLD signal intensity over time while two of the eight HOMzQ175 mice showed no activation. A repeated measures one-way ANOVA showed no significant main effect for genotype (*p* = 0.067) but did show a differential effect of genotype on BOLD response following presentation of almond odor [genotype × time: *F*_(2,138)_ = 1.45 *p* = 0.0007].

**Figure 8:**
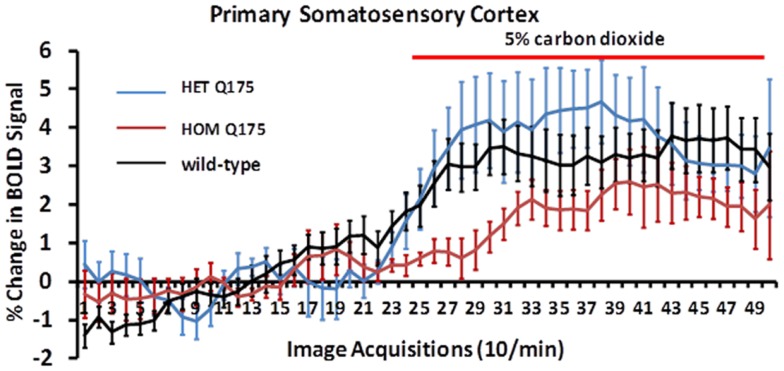
**Blood oxygen level dependent signal change to carbon dioxide challenge**. Shown are time-course data for each genotype for the percentage change in BOLD signal intensity in the somatosensory cortex in response to the challenge of 5% carbon dioxide. Each image acquisition is the mean ± SEM.

Shown in Figure 8 are changes in BOLD signal over time in response to 5% CO_2_ challenge for wild-type (*n* = 7), HETzQ175 (*n* = 7), and HOMzQ175 (*n* = 10) mice. For both wild-type and HETzQ175 conditions, one out of the seven animals did not response to CO_2_ challenge, i.e., no activated voxels in the primary somatosensory cortex, while 4 out of the 10 animals in the HOMzQ175 genotype did not respond to CO_2_ challenge. A repeated measures one-way ANOVA demonstrated that the time-course of BOLD responses to CO_2_ differed by genotype [genotype × time: *F*_(2,98)_ = 1.65, *p* = 0.001]. *Post hoc* pairwise comparisons at each repetition indicated that the differential effect of genotype on BOLD response occurred in the first five repetitions (30 s) following onset of the CO_2_ challenge. HOMzQ175 rats displayed diminished BOLD responses compared to both wild-type and HETzQ175 rats during repetitions 26–30 (*p* < 0.05 for all comparisons).

Shown in Table 2 are the comparisons in brain volumes across wild-type, HET, and HOMzQ175 mice. The brain areas are rank order for their significance and are truncated from a larger list of 116 regions of activation (for complete list see Table S2 in Supplementary Material). The multiple comparison analysis showed 46 brain areas to differ significantly in volume across phenotypes. *Post hoc* analysis using Fisher’s PLSD showed that the significant differences (*p* < 0.05) were all between the HET and HOMzQ175 mice and not between wild-type vs. HET or wild-type vs. HOMzQ175 mice.

## Discussion

We chose to challenge wild-type, HET, and HOMzQ175 mice with the smell of almond as a means of differentiating their brain activity toward a stimulus with a high emotional and motivational valence. In human and animal fMRI studies, desirable foods have been identified as motivating/rewarding stimuli activating limbic areas, particularly the orbital frontal, cingulate and insular cortices, amygdala, and striatum (10, 19–22). In a recent study, we were able to show that rats possess an innate sensitivity to energy rich food (such as almonds) and that this results in neural activation of the olfactory system and its connections to other brain areas (10). In a pilot study using serial dilutions of 100% benzaldehyde (almond odor), we identified a threshold dilution (1/10,000 v/v) that gave a robust and consistent pattern of brain activity to wild-type and HET Q175 mice, while the responsiveness of HOM Q175 was less (Figures 6 and 7) particularly in the glomerular layer of the olfactory bulb (Figure 4; Table 1).

Loss in olfactory discrimination and detection sensitivity are common with HD (23–26) and may appear prior to significant motor or cognitive dysfunction (27). Individuals at risk for HD, show a dysfunction in source memory for olfaction, i.e., the context associated with the memory (28). Transgenic mouse models of HD shows micro-aggregates of huntingtin proteins in primary olfactory system (29) and a reduction in structural neuroplasticity in olfactory cortex that may be causally related to the impairment in olfactory memory (24). These abnormalities may have contributed to the functional differences observed in the present study although imaging these phenotypes at 1 year of age preclude us from drawing any conclusions about olfaction as a biomarker of pre-symptomatic HD.

The imaging data suggest a “gene-dose effect,” i.e., HETzQ175 mice show a reduction in brain activity from wild-type that is further reduced in HOMzQ175 mice. Indeed, of the 17 brain areas identified as responding differently to almond odor across genotypes, HOMzQ175 showed the greatest differences between wild-type and HETzQ175. Since the HETzQ175 form of HD is most prevalent in the population and is characterized by slow disease progression transitioning from a pre-symptomatic to symptomatic phenotype, greater emphasis is placed on understanding the HETzQ175 genotype. Only 6 out of 17 areas identified in Figure 3 were different between wild-type and HETzQ175 mice. These included the frontal association cortex, anterior cingulate cortex, subiculum of the hippocampus, lateral posterior thalamus and vestibular area, and locus coeruleus of the medulla oblongata. None of these areas were different between HETzQ175 and HOMzQ175 mice, so they represent brain areas deviating from the norm during disease progression and antecedent to any HD phenotype in the HETzQ175 mouse model (6, 7). Interestingly, the frontal association and cingulate cortices were shown to have reduced metabolic activity based on 2DG autoradiography in the R6/2 mouse model of HD (30).

In HD, striatal and cortical atrophy are the most common findings, and they correlate with cognitive deficits in attention, working memory, and executive functions (2). Cognitive decline is a well documented sign of early HD (31) even affecting individual at risk for HD (32–34). A time-dependent and gene-dose dependent change in cognitive function is a common feature of transgenic mouse models of HD (6, 7, 35–38). In wild-type mice, activation of subcortical and cortical brain areas identified with quantitative autoradiography for 2DG involve time-dependent changes in brain activity associated with memory consolidation. These brain areas include frontal association cortex, anterior cingulate, sensory motor cortices together with thalamus and hippocampus (39). All of these areas are represented in the brain map shown in Figure 3 reflecting areas differing in odor-induced activation across genotypes. Transgenic mouse models of HD suggest dysfunction in hippocampal dependent short term memory (40) and increased activity in local field potentials in the subiculum, dentate gyrus, and temporal cortex with an enhanced susceptibility to autogenic seizures (30). Neurotransmission between cortical and subcortical areas is affected in HD. Alterations in dendritic spine survival and density in cortical neurons characteristic of mouse model of HD accompany early symptoms of HD (41). There is also evidence that altered excitatory and inhibitory inputs to pyramidal neurons in the cortex are characteristics of disease progression in various mouse models of HD, which points to early signs of synaptic dysregulation involving glutamate and GABA signaling (42, 43).

Changes in brain morphology and reduced brain volume, particularly in the area of the striatum and cortex are common features of disease progression in HD (44–46). Studies in mouse models of HD mice also report significant volume loss in the striatum, and neocortex as compared to wild-type (30, 47–49). In our studies, the use of the 3D segmented atlas to calculate the average volume for 116 different brain areas across wild-type, HETzQ175, and HOMzQ175 mice corroborated what has been reported by Heikkinen and coworkers for striatal volumes across genotypes in zQ175 mice (7). At 10 months of age HOMzQ175 mice show a striatal volume (caudate/putamen) of just under 16 mm^3^ as compared to a wild-type of volume of just under 19 mm^3^, a decrease of ca. 15%. In these studies, we report striatal volume of ca. 15 mm^3^ for HOMzQ175 and ca. 18 mm^3^ for wild-type or about a 17% reduction in volume. The major difference between studies is that we did not observe a reduction in ROI volumes in HETzQ175 mice. Indeed, there were no significant differences in ROI volumes between wild-type and HETzQ175 mice.

One of the more interesting aspects of this study is the presentation of almond odor, to “odor naïve” animals differing only in the protein expression of the single *huntingtin* gene. From 116 different brain areas, only 17 were found to differ in their activity when comparing wild-type, HETzQ175, and HOMzQ175 genotypes as shown in Figure 3. When viewed in the context of an integrated neural circuit, the areas of activation do not comprise a distinguishing “finger print” of brain function, e.g., motivation and reinforcement, pain, fear, or any particular neurochemical signaling pathway like dopamine or serotonin. Indeed, the caudate/putamen, thalamus, CA3 and CA1 of the hippocampus and much of the cortex are targeted areas in HD showing dramatic changes in function and morphology with disease progression. Yet, these areas were not identified with this almond odor provocation paradigm. What then is the connection, if any, between the glomerulus of the olfactory bulb, frontal association cortex, and anterior cingulate in the rostral part of the brain to the vestibular area, spinal trigeminal nucleus, and locus coeruleus in the most caudal part of the brain and the hypothalamus and amygdala in between? The answer may be found in a study by Fujinaga and coworkers mapping the neuroanatomical distribution of HAP1 mRNA in the male mouse brain (50). HAP1 can complex with Htt protein in the cytoplasm of neurons. Together, they regulate autophagosome transport, bidirectional movement of vesicles along the neuron axis involved in degradation of cellular debris (51). The presence of mutant HTT protein or reduction in HAP1 can impair the Htt/HAP1 interaction and disrupt autophagosome transport potentially leading to neuronal death (51). However, there are multiple lines of evidence that HAP1 is not contributing to the neuropathology of mutant Htt (52–54); instead, mutant Htt may be impacting the normal function of HAP1 (55, 56). To this point, HAP1 is not found in CA3/CA1 of hippocampus, thalamus, or much of the cortical mantel with only small amounts in the somatosensory cortex and caudate/putamen. Instead, its highest concentrations are found in a majority of those areas identified in Figure 3. Hence, the brain activity across wild-type, HETzQ175, and HOMzQ175, differentiated by the odor of almond, maps onto the neuroanatomical localization of HAP1. Might there be some functional relevance to this observation?

While HAP1 has been shown to function in intracellular trafficking of pro-brain-derived-neurotrophic factor (57, 58), epidermal growth factor (59), and gamma-aminobutyric acid type A receptor (60), to note a few, it also directly impacts animal behavior via the hypothalamus. It was proposed by Chan and coworkers that this putative neural circuit connected by HAP1 expressing neurons functions in the control of instinctual behaviors, e.g., feeding, sex, and aggression, that involve autonomic and neuroendocrine processes (53). This notion was supported by the finding that transgenic mice deficient in HAP1, suffer from postnatal malnutrition and morbidity because of a dysfunction in feeding behavior. The link between HAP1 and feeding behavior was subsequently corroborated by others (56, 61). Given the odor stimulant used in the present study (almond) is intrinsically rewarding, there may be a causal link between the pattern of BOLD activation that differentiates the wild-type and HD genotypes, to the neuroanatomical localization of HAP1.

### Caveats and data interpretation

For any imaging study on awake animals, the issues and consequences related to the stress of head restraint and restricted body movement must be considered. Protocols have been developed to help lessen the stress of an imaging study by acclimating animals to the environment of the MR scanner and the restraining devices helping to reduce stress hormones levels and measures of sympathetic autonomic activity (12, 62). These acclimation procedures put animals through several simulated imaging sessions and have been used to study sexual arousal in monkeys (63), generalized seizures in rats and monkeys (64, 65), and exposure to psychostimulants like cocaine (66–68), nicotine (69), and apomorphine (62, 70). Nonetheless, one must consider the experimental confound that exists with low levels of arousal and stress associated with imaging awake animals. Indeed, the different phenotypes in this study may have very different sensitivities to restraint stress.

Another consideration when interpreting the data is the morphological changes in brain structure that occur over time in wild-type, HET, and HOMzQ175 mice as noted above. This raises the possibility that regional differences in brain volume in HOMzQ175 may have influenced the BOLD signal analysis particularly when the data is reported as volume of activation, i.e., number of voxels activated in a 3D brain volume. To control for this possibility, we normalized the volume of activation to the brain volume of interest for each subject prior to statistical comparisons for all three genotypes.

The data in Figure 8 clearly show that the HOMzQ175 have diminished vascular responsivity to CO_2_ challenge. This raises the possibility that the blunted BOLD response in HOMzQ175 mice may be due to ineffective coupling of blood/flow and metabolism. Cepeda-Prado and coworkers (30) using measures of cerebral blood volume (CBV) correlated with neuronal excitability, suggest there is normal neurovascular coupling in HD mice, but a paradoxical decrease in metabolism with very high CBV suggestive of impaired neurometabolic coupling. The implications are far reaching and may underscore a mechanism that contributes to loss of cognitive function with disease severity. There are no reports in the human or animal imaging literature that we know of that show challenges with CO_2_.

### Summary

The prospective capability of animal imaging to follow changes in brain neurobiology following genetic or environmental insult has great value in the field of HD research as one can follow the etiology and pathophysiology of disease progression. In addition, the combination of awake fMRI in mice with an imaging genetics approach (71) represents a powerful experimental strategy that permits the identification of the affect of single gene mutations on neural circuits regulating emotion and cognition. While imaging genetics in humans takes advantage of natural polymorphisms to examine the genetic basis of differences in neural activation, imaging genetics in transgenic animals is a more targeted approach that offers the potential to investigate the contribution of a single gene to neural response patterns. When this neural activity is combined with a 3D segmented, annotated MRI mouse atlas it is possible to reconstruct distributed integrated neural circuits both in 3D and 2D that “finger print” the pattern of brain activity to a provocation paradigm as is the case here.

## Conflict of Interest Statement

Craig Ferris, Mark Nedelman, and Praveen Kulkarni have a financial interest in Ekam Imaging and Ekam Solutions, the company that sponsored the research and developed the 3D segmented annotated mouse atlas. Craig Ferris and Steven Toddes have a financial interest in Animal Imaging Research, the company that makes the awake mouse imaging system. The other co-authors declare no conflicts of interest.

## Supplementary Material

The Supplementary Material for this article can be found online at http://www.frontiersin.org/Journal/10.3389/fneur.2014.00094/abstract
