# Supplementary material for: Studies on the Q175 Knock-in Model of Huntington’s Disease Using Functional Imaging in Awake Mice: Evidence of Olfactory Dysfunction
Source: Front Neurol. 2014 Jun 30;5:94. doi: 10.3389/fneur.2014.00094 (PMC4074991; doi:10.3389/fneur.2014.00094)
Supplement: Supplementary file 1 [file Data_Sheet_1.ZIP › Table S1.PDF]

Changes in Positive BOLD Signal in Response to Odor of Almond

Region of Interest(ROI)	Wild-Type			Heter zQ175			Homo zQ175			
	Med	Max	Min	Med	Max	Min	Med	Max	Min	P value
medial amygdaloid area	4	20	0	2	10	0	0	0	0	0.002
ventral medial hypothalamic area	7	68	0	13	33	0	0	0	0	0.004
frontal association ctx	10	23	3	0	14	0	0	11	0	0.006
subiculum	3	27	0	1	4	0	0	2	0	0.008
anterior hypothalamic area	3	27	0	4	14	0	0	0	0	0.008
vestibular area	8	62	0	0	8	0	0	3	0	0.009
endopiriform area	4	21	0	0	3	0	0	0	0	0.011
extended amydala	1	11	0	0	3	0	0	0	0	0.012
dentate gyrus	6	32	0	1	7	0	0	4	0	0.016
anterior cingulate area	4	28	0	0	8	0	0	3	0	0.026
lateral caudal hypothalamic area	4	39	0	4	25	0	0	11	0	0.026
lateral posterior thalamic area	3	27	0	0	0	0	0	33	0	0.027
primary somatosensory ctx	4	21	2	4	18	0	0.5	5	0	0.03
secondary somatosensory ctx	1	14	0	3	6	1	0	4	0	0.032
ventral tegmental area	0	28	0	0	3	0	0	0	0	0.043
glomerular layer	6	29	1	3	40	1	0.5	10	0	0.044
locus ceruleus	20	50	0	0	31	0	0	33	0	0.045
lemniscal area	0	52	0	0	0	0	0	0	0	0.047
reuniens thalamic area	0	0	0	0	10	0	0	0	0	0.047
principal sensory nucleus trigeminal	1	21	0	1	6	0	0	1	0	0.048
medial mammillary area	23	82	0	3	31	0	0	35	0	0.059
lateral rostral hypothalamic area	1	12	0	2	12	0	0	8	0	0.068
lateral lemniscus	3	20	0	0	3	0	0	4	0	0.073
anterior amygdaloid area	5	42	0	0	14	0	0	2	0	0.077
parietal ctx	7	35	0	0	67	0	0	24	0	0.08
pituitary	7	83	0	0	12	0	0	33	0	0.083
superior colliculus	1	20	0	1	7	0	0	4	0	0.088
spinal trigeminal nuclear area	9	41	0	1	18	0	0.5	5	0	0.093
substantia nigra	3	18	0	3	9	0	0	5	0	0.094
orbital ctx	3	14	0	1	5	0	0.5	2	0	0.095
posterior hypothalamic area	0	41	0	0	32	0	0	0	0	0.099
secondary motor ctx	5	28	0	3	28	0	1	7	0	0.105
central amygdaloid area	4	26	0	1	16	0	0	8	0	0.11
olfactory tubercles	13	29	0	4	29	0	0	10	0	0.114
anterior olfactory area	2	20	0	1	15	0	0	13	0	0.114
simple lobule cerebellum	4	45	0	3	25	0	0.5	7	0	0.118
prelimbic ctx	5	23	0	0	11	0	0	7	0	0.123
primary motor ctx	4	19	2	4	19	1	0	13	0	0.124

pedunculopontine tegmental area	6	41	0	1	8	0	4	24	0	0.129
2nd cerebellar lobule	0	18	0	0	2	0	0	3	0	0.132
retrosplenial rostral ctx	6	31	2	1	53	0	2	12	0	0.14
cortical amygdaloid area	2	21	0	2	11	0	0	5	0	0.142
habenular area	0	67	0	0	0	0	0	18	0	0.142
granular cell layer	1	24	0	2	41	0	0	12	0	0.143
claustrum	0	29	0	0	67	0	0	0	0	0.153
insular rostral ctx	2	24	0	2	3	0	0	3	0	0.172
inferior colliculus	5	36	0	9	32	1	3	10	0	0.186
medial preoptic area	2	24	0	2	15	0	0	7	0	0.19
cerebellar nuclear area	0	8	0	0	12	0	0	0	0	0.194
lateral geniculate	2	17	0	0	4	0	0	15	0	0.207
reticulotegmental nucleus	0	18	0	1	7	0	0	18	0	0.214
retrosplenial caudal ctx	14	27	2	3	27	1	2.5	25	0	0.214
dorsal medial hypothalamic area	0	32	0	0	53	0	0	0	0	0.217
lateral amygdaloid area	9	64	0	0	17	0	0	23	0	0.217
flocculus cerebellum	6	31	3	3	18	0	1.5	11	0	0.231
entorhinal ctx	5	30	1	3	8	0	2.5	8	0	0.237
zona incerta	0	19	0	0	2	0	0	20	0	0.239
4th cerebellar lobule	0	39	0	0	8	0	0	1	0	0.239
olivary complex	0	3	0	0	0	0	0	4	0	0.259
caudate putamen	3	19	0	1	12	1	1	10	0	0.266
lateral paragigantocellular area	0	19	0	0	2	0	0	3	0	0.269
intermediate reticular area	0	2	0	0	9	0	1	8	0	0.276
bed nucleus stria terminalis	5	12	0	3	7	0	4	8	0	0.276
rostral piriform ctx	2	23	0	1	4	0	0	3	0	0.279
dorsal raphe	0	31	0	0	7	0	0	8	0	0.316
periaqueductal gray	3	35	0	1	16	0	0	4	0	0.317
3rd cerebellar lobule	0	37	0	0	2	0	0	2	0	0.325
central medial thalamic area	0	0	0	0	38	0	0	5	0	0.338
insular caudal ctx	1	32	0	0	13	0	0	2	0	0.351
posterior thalamic area	2	20	0	0	10	0	0	27	0	0.366
parabrachial area	0	21	0	0	21	0	0	10	0	0.367
paraventricular hypothalamic area	0	29	0	0	33	0	0	0	0	0.375
gigantocelllaris reticular area	0	6	0	1	2	0	0	6	0	0.396
CA3 hippocampus	2	23	0	2	4	0	1	10	0	0.407
lateral preoptic area	0	35	0	0	7	0	0	18	0	0.41
diagonal band of Broca	0	41	0	0	11	0	0	3	0	0.414
infralimbic ctx	0	22	0	0	7	0	0	30	0	0.42
ventral thalamic area	1	20	0	0	4	0	0	3	0	0.434
accumbens shell	0	18	0	0	4	0	0	1	0	0.434

visual 1 ctx	6	39	1	9	50	0	3.5	26	0	0.439
medial dorsal thalamic area	0	28	0	2	22	0	0	13	0	0.443
pontine area	1	25	0	0	4	0	0	6	0	0.466
tenia tecta ctx	1	40	0	2	44	0	0	3	0	0.486
pontine reticular nucleus caudal	0	12	0	3	5	0	2	16	0	0.503
crus of ansiform lobule	5	29	0	10	29	0	3	13	0	0.509
medial geniculate	2	37	0	1	6	0	0	17	0	0.509
ventral pallidum	0	17	0	1	6	0	0	3	0	0.519
auditory ctx	2	9	0	3	13	0	2	13	0	0.542
temporal ctx	5	18	0	3	32	0	0	21	0	0.552
anterior thalamic area	2	23	0	0	7	0	2	12	0	0.573
parafascicular thalamic area	0	36	0	0	20	0	0	8	0	0.584
medial septal area	0	31	0	0	21	0	0	25	0	0.587
anterior pretectal thalamic area	0	58	0	0	2	0	0	28	0	0.595
lateral dorsal thalamic area	2	18	0	0	7	0	0	24	0	0.629
mesencephalic reticular formation	2	16	0	2	5	0	0	6	0	0.634
parvicellular reticular area	0	15	0	1	20	0	0.5	9	0	0.638
CA1 hippocampus	1	13	0	1	2	0	1	3	0	0.652
interpeduncular area	0	18	0	0	20	0	0	9	0	0.66
basal amygdaloid area	3	13	0	1	10	0	1.5	6	0	0.71
reticular thalamic area	2	8	0	1	8	0	0	9	0	0.74
accumbens core	1	10	0	0	3	0	0	6	0	0.756
median raphe area	2	20	0	1	9	0	2	27	0	0.796
globus pallidus	2	4	0	1	5	0	1	11	0	0.823
lateral septal area	3	24	0	3	22	0	7	20	0	0.826
caudal piriform ctx	3	24	0	2	8	0	6.5	24	0	0.895
pontine reticular nucleus oral	1	6	0	1	4	0	1	6	0	0.904
paraventricular thalamic area	0	35	0	0	9	0	0	9	0	0.958
facial nucleus	0	19	0	0	15	0	0	5	0	0.981
