# Supplementary material for: Studies on the Q175 Knock-in Model of Huntington’s Disease Using Functional Imaging in Awake Mice: Evidence of Olfactory Dysfunction
Source: Front Neurol. 2014 Jun 30;5:94. doi: 10.3389/fneur.2014.00094 (PMC4074991; doi:10.3389/fneur.2014.00094)
Supplement: Supplementary file 1 [file Data_Sheet_1.ZIP › Table S2.PDF]

Region of Interest(ROI)	Wild-Type			Heter zQ175			Homo zQ175			
	X Vol mm^3	X Vox #	SE	X Vol mm^3	X Vox #	SE	X Vol mm^3	X Vox #	SE	P value
caudate putamen	17.6	1171	102.9	19.6	1304	93.1	14.8	987	93.8	0.03
caudal piriform ctx	5.9	392	35.7	6.5	435	31.5	4.9	325	33.5	0.03
endopiriform area	1.0	67	7.6	1.2	78	6.5	0.8	54	6.4	0.03
anterior cingulate area	2.8	186	18.5	3.2	213	16.3	2.4	158	16.6	0.03
fimbria hippocampus	1.8	123	11.8	2.1	139	10.3	1.5	102	11.4	0.03
globus pallidus	2.7	180	18.0	3.0	201	15.1	2.2	147	15.4	0.03
primary somatosensory ctx	20.7	1381	127.2	22.9	1528	109.8	17.4	1158	116.0	0.03
superior colliculus	8.3	551	48.4	9.3	618	43.3	6.9	458	43.7	0.03
median raphe area	1.3	86	7.2	1.5	97	6.8	1.1	73	6.2	0.03
substantia nigra	1.6	109	10.1	1.8	119	7.8	1.4	90	9.0	0.03
parietal ctx	0.5	31	2.6	0.6	37	3.4	0.3	23	3.1	0.03
prelimbic ctx	1.7	112	10.9	1.9	126	9.3	1.4	96	9.8	0.03
CA1 hippocampus	8.0	536	47.6	8.9	590	42.2	6.8	454	43.4	0.03
entorhinal ctx	16.0	1066	99.1	17.7	1180	82.2	13.5	903	93.8	0.04
olfactory tubercles	1.8	120	11.5	2.0	136	11.5	1.5	99	10.4	0.04
ventral medial hypothalamic area	0.7	49	4.6	0.9	57	4.4	0.6	39	4.3	0.04
medial geniculate	1.3	87	8.3	1.5	97	6.6	1.1	72	6.7	0.04
retrosplenial caudal ctx	4.3	285	25.4	4.7	312	23.4	3.5	233	24.2	0.04
ventral pallidum	1.9	128	13.2	2.2	144	11.5	1.6	107	11.7	0.04
auditory ctx	3.8	256	24.2	4.4	290	20.4	3.2	216	23.0	0.04
medial amygdaloid area	2.0	132	12.7	2.2	149	11.2	1.7	111	10.7	0.04
dentate gyrus	6.3	420	38.4	6.9	460	34.0	5.2	347	37.4	0.04
lateral geniculate	0.7	46	4.2	0.8	52	4.1	0.5	36	3.7	0.04
basal amygdaloid area	3.1	206	20.2	3.5	230	18.2	2.6	173	17.8	0.04
secondary somaotsensory ctx	5.6	370	34.1	6.1	407	30.9	4.6	308	31.5	0.04
lateral rostral hypothalamic area	2.9	191	17.3	3.2	214	15.3	2.4	160	17.1	0.04
periaqueductal gray	5.3	352	32.2	5.8	388	28.9	4.4	296	28.5	0.04
central amygdaloid area	1.8	122	11.4	2.0	133	9.7	1.5	103	10.3	0.04
anterior hypothalamic area	2.3	152	13.1	2.5	169	11.3	2.0	131	12.1	0.04
insular rostral ctx	5.9	392	34.4	6.5	431	31.6	4.9	326	32.7	0.04
mesencephalic reticular formation	6.9	458	42.0	7.5	503	34.3	5.7	383	37.3	0.04
ventral tegmental area	0.6	39	4.4	0.6	42	2.9	0.5	31	3.5	0.04
subiculum	7.2	478	44.3	7.9	525	37.4	6.0	399	39.1	0.04
ventral thalamic area	4.9	328	28.6	5.5	364	26.9	4.2	278	27.5	0.04
inferior colliculus	6.5	436	40.8	7.2	482	34.2	5.6	370	36.6	0.05
orbital ctx	5.5	367	33.2	6.1	407	29.6	4.7	311	30.4	0.05
CA3 hippocampus	3.4	225	21.9	3.8	251	19.1	2.8	185	20.1	0.05
rostral piriform ctx	8.1	540	47.9	8.9	590	42.8	6.7	449	47.0	0.05
visual 1 ctx	13.6	909	82.6	14.7	978	69.4	11.6	773	75.9	0.05
dorsal raphe	0.6	37	3.5	0.6	40	3.0	0.5	31	3.0	0.05
primary motor ctx	5.1	337	32.6	5.7	377	30.6	4.3	287	31.7	0.05
paraventricular hypothalamic area	0.2	10	1.4	0.2	12	1.1	0.1	8	1.2	0.05
pituitary	0.9	57	6.4	1.0	68	5.4	0.7	48	5.4	0.06
central medial thalamic area	0.6	40	3.5	0.6	43	3.6	0.5	30	3.3	0.06
ventricle	5.6	371	37.0	6.1	408	31.3	4.8	322	35.1	0.06
insular caudal ctx	2.4	157	15.3	2.5	168	12.2	1.9	128	13.7	0.06
cortical amygdaloid area	4.1	275	25.6	4.5	299	21.3	3.5	234	24.6	0.06
anterior pretectal thalamic area	0.9	60	7.6	1.1	71	6.4	0.8	50	5.5	0.06
tenia tecta ctx	1.6	104	9.9	1.7	116	8.0	1.3	89	8.9	0.06

reticulotegmental nucleus	1.0	67	7.2	1.1	73	6.2	0.8	53	5.4	0.06
reticular thalamic area	1.5	100	9.7	1.7	110	9.5	1.2	81	8.9	0.06
flocculus cerebellum	4.1	274	26.8	4.4	294	21.1	3.3	223	24.7	0.07
2nd cerebellar lobule	1.8	121	12.5	2.1	139	9.4	1.5	101	11.6	0.07
posterior thalamic area	1.0	66	7.1	1.1	73	4.8	0.8	55	6.7	0.07
anterior thalamic area	1.5	97	9.1	1.6	106	7.5	1.2	82	8.8	0.07
secondary motor ctx	5.4	362	34.1	5.8	389	28.9	4.5	301	30.6	0.07
anterior olfactory area	7.3	484	43.5	7.8	520	46.3	6.1	404	41.6	0.07
granular cell layer	6.1	409	39.7	6.0	401	47.9	4.9	327	35.8	0.07
medial mammillary area	0.8	54	4.9	0.9	59	4.6	0.7	45	4.7	0.08
parafascicular thalamic area	0.2	12	1.2	0.2	16	1.8	0.2	11	1.1	0.08
lateral septal area	2.4	158	13.6	2.6	170	11.9	2.0	131	13.7	0.08
dorsal hippocampal commissure	0.4	25	3.4	0.4	26	2.7	0.3	19	3.3	0.08
pontine area	2.5	164	16.2	2.7	177	13.7	2.0	136	16.3	0.08
anterior commissure	0.3	20	2.8	0.4	26	2.7	0.2	15	3.5	0.08
retrosplenial rostral ctx	4.2	279	26.1	4.4	295	21.8	3.5	233	23.3	0.08
frontal association ctx	3.6	239	20.9	3.8	250	17.2	2.9	191	20.0	0.08
accumbens core	1.3	86	7.8	1.4	92	6.4	1.1	72	7.2	0.08
accumbens shell	1.5	98	9.5	1.5	103	7.5	1.2	82	8.2	0.09
cerebral peduncle	4.9	324	31.4	5.7	377	30.2	4.6	306	31.7	0.09
anterior amygdaloid area	0.8	54	5.0	0.9	61	4.2	0.7	44	5.2	0.10
reuniens thalamic area	0.6	40	4.0	0.6	43	3.2	0.5	33	3.7	0.10
external capsule	1.8	117	17.1	1.9	126	14.9	1.3	86	16.4	0.10
bed nucleus stria terminalis	1.5	100	8.5	1.6	108	8.7	1.3	85	7.8	0.10
temporal ctx	1.7	114	10.8	1.8	122	9.8	1.4	96	10.3	0.11
lateral preoptic area	0.4	29	2.9	0.5	32	3.2	0.3	22	2.0	0.11
habenular area	0.2	16	1.4	0.3	18	1.5	0.2	13	1.7	0.11
glomerular layer	8.3	552	54.7	8.0	534	69.5	6.7	447	51.6	0.11
extended amydala	0.8	54	5.4	0.9	58	4.1	0.7	46	5.3	0.12
dorsal medial hypothalamic area	0.3	23	2.4	0.4	24	2.2	0.3	18	2.1	0.12
4th cerebellar lobule	1.0	65	6.5	1.1	75	6.5	0.9	57	6.5	0.12
parabrachial area	0.6	40	3.9	0.7	44	3.5	0.5	35	3.6	0.13
lateral amygdaloid area	0.6	39	4.5	0.6	43	3.8	0.5	32	3.8	0.13
interpeduncular area	0.5	34	3.3	0.6	37	2.7	0.4	29	3.3	0.14
medial septal area	0.3	19	1.8	0.3	21	2.1	0.2	16	1.8	0.14
crus of ansiform lobule	4.8	321	57.3	6.6	440	50.4	7.3	485	46.0	0.15
intermediate reticular area	1.0	66	8.1	1.2	81	10.7	1.5	103	10.6	0.15
medial dorsal thalamic area	0.7	44	4.8	0.7	48	3.6	0.6	38	4.5	0.16
lemniscal area	0.8	53	5.8	0.9	59	6.0	0.7	44	5.5	0.16
medial preoptic area	1.4	96	9.2	1.6	109	10.1	1.2	79	8.0	0.17
pontine reticular nucleus caudal	4.3	285	26.7	4.2	281	25.1	3.3	220	27.1	0.18
lateral posterior thalamic area	0.8	52	5.5	0.9	57	4.4	0.7	46	4.9	0.18
optic tract	0.3	19	2.5	0.3	21	1.8	0.2	16	2.4	0.19
lateral lemniscus	0.8	54	6.4	0.9	58	5.2	0.7	44	6.3	0.19
internal capsule	1.8	122	11.9	1.9	129	11.1	1.5	103	11.0	0.21
lateral caudal hypothalamic area	1.2	81	7.7	1.3	89	9.0	1.0	69	6.9	0.21
diagonal band of Broca	0.9	58	6.6	0.9	61	5.2	0.7	49	5.8	0.21
paraventricular thalamic area	0.5	32	3.4	0.5	35	3.1	0.4	28	2.8	0.22
spinal trigeminal nuclear area	2.0	131	15.0	2.4	160	22.3	2.9	191	19.9	0.23
posterior hypothalamic area	0.3	20	2.0	0.4	25	2.6	0.3	18	2.7	0.24
olivary complex	0.7	48	4.9	0.7	47	4.1	0.6	39	5.3	0.24
fornix	0.1	9	1.5	0.1	8	1.0	0.1	6	1.0	0.27

lateral dorsal thalamic area	0.9	60	5.3	0.9	62	5.9	0.8	50	5.4	0.31
gigantocelllaris reticular area	3.5	235	23.4	4.1	270	34.2	4.6	307	31.4	0.33
parvicellular reticular area	1.2	77	8.3	1.3	86	9.9	1.5	97	10.5	0.34
cerebellar nuclear area	1.0	68	10.1	1.3	87	9.6	1.2	82	9.7	0.35
locus ceruleus	0.1	7	1.2	0.1	8	1.2	0.1	6	1.2	0.42
lateral paragigantocellular area	1.0	64	9.2	1.0	66	11.1	1.2	80	8.7	0.51
vestibular area	2.0	133	14.2	2.4	157	15.9	2.2	148	13.5	0.62
infralimbic ctx	0.3	21	3.4	0.4	24	3.1	0.3	20	2.4	0.63
stria medullaris	0.1	8	1.7	0.1	8	1.5	0.1	6	1.4	0.72
claustrum	0.1	5	1.4	0.1	5	1.0	0.1	4	0.8	0.77
transverse fibers pons	0.0	1	0.5	0.0	1	0.4	0.0	1	0.2	0.79
pyramidal tracts	0.9	61	5.0	1.0	66	5.4	0.9	62	6.3	0.89

X^-
